# Large Anthrax Outbreak in a New Locality in Croatia, 2022

**DOI:** 10.3390/life14030349

**Published:** 2024-03-07

**Authors:** Ljiljana Žmak, Selma Bošnjak, Iva Pem Novosel, Tajana Juzbašić, Tatjana Vilibić-Čavlek, Irena Tabain, Tihana Miškić, Ivana Lohman Janković, Inoslav Brkić, Ana Gverić Grginić

**Affiliations:** 1 Department of Microbiology, Croatian Institute of Public Health, 10000 Zagreb, Croatia; selma.bosnjak@hzjz.hr (S.B.); tajana.juzbasic@hzjz.hr (T.J.); tatjana.vilibic-cavlek@hzjz.hr (T.V.-Č.); irena.tabain@hzjz.hr (I.T.); ana.gveric-grginic@hzjz.hr (A.G.G.); 2 Department of Microbiology, School of Medicine, University of Zagreb, 10000 Zagreb, Croatia; 3 Department of Communicable Diseases Epidemiology, Croatian Institute of Public Health, 10000 Zagreb, Croatia; iva.pem-novosel@hzjz.hr; 4 Veterinary and Food Safety Directorate, Ministry of Agriculture, 10000 Zagreb, Croatia; tihana.miskic@mps.hr (T.M.); ivana.lohman@mps.hr (I.L.J.); 5 Public Health Institute of Sisak-Moslavina County, Kralja Tomislava 1, 44000 Sisak, Croatia; uprava@zzjz-sk.hr

**Keywords:** anthrax, zoonosis, one health, emerging diseases

## Abstract

*Bacillus anthracis* is a well-known zoonotic pathogen that can cause disease in both animals and humans. Moreover, it has a high bioterrorism potential as its lethal spores are resistant to inactivation, are easy to produce in large quantities, and are easily spread over large areas. Anthrax cases occur in different parts of the world, including most European countries. Specific areas of Croatia are long known as anthrax districts, but with sporadically reported cases over the years. Here, we present a major outbreak of animal and human anthrax in the region of Lonjsko Polje in Croatia, a region not known to have anthrax cases in the past. The outbreak started in July 2022 and lasted several months, but most human and animal cases were reported in the first month. During the outbreak, there were 17 reported human cases of cutaneous anthrax and 29 laboratory-confirmed animal cases. However, due to issues in reporting in animals and the late finding of the carcasses, which made laboratory diagnostics challenging, the actual number of animal cases was probably significantly higher.

## 1. Introduction

Anthrax is a zoonosis that has been present for thousands of years in different parts of the world. Until the implementation of the vaccine in the 1930s, this disease was one of the most important causes of mortality in cattle, sheep, and goats, with zoonotic transmission to humans [1]. The causative agent, *Bacillus anthracis*, is the first bacterium that was discovered by the microbiologist Robert Koch in 1876. The peculiarity of this bacterium is that it has two life forms, the vegetative bacillary form and the dormant spore form, which can survive in the environment for decades [2]. The vegetative form is present within a suitable environment rich with nutrients (mammalian body), while the spores are formed in aerobic microenvironments with limited nutrients (environment). Humans and animals become infected through contact with anthrax spores, either by inhalation and ingestion or through skin exposure [3]. Depending on the mode of infection, several clinical presentations may occur: cutaneous, gastrointestinal, and inhalation disease. The cutaneous form represents more than 95% of all anthrax cases worldwide and is mainly acquired by direct contact with sick animals or contact with contaminated animal hides, hair, bone products, or wool. The spores have a low invasive potential, so cutaneous entry is possible through non-intact skin, typically in areas with cuts or abrasions. There are also sporadic reports of transmission by insect bites [4]. The cutaneous form usually develops 2–5 days (range 1–7 days) postexposure, most commonly on exposed skin areas, such as forearms and shins. The characteristic cutaneous presentation is a painless, black necrotic lesion with a round raised edge [5]. The gastrointestinal and pulmonary forms of the disease are rare and often fatal, especially in the case of pulmonary involvement. Due to its rarity, there should always be a concern about the possibility of a bioterrorist attack when more cases of inhalation anthrax occur. The last two outbreaks of pulmonary anthrax were both of non-natural occurrence [6]. Namely, the outbreak of inhalation anthrax in the Russian city of Sverdlovsk in 1979 was caused by an unintentional release of anthrax spores from a Soviet bioresearch facility causing 96 human cases of inhalation anthrax. Moreover, the last outbreak of inhalation anthrax occurred as a result of a terroristic attack in the USA in 2001 [6].

Unlike inhalation anthrax, the cutaneous form is linked to contact with sick domestic and wild animals, and globally there are around 2000 human anthrax cases per year [7]. Although anthrax may occur in many European countries, the incidence is extremely low. Namely, in 2021 in all the European Union/European Economic Area (EU/EEA) countries, there were only seven (confirmed and probable) reported cases of anthrax from three countries, including Bulgaria, France, and Spain [8]. In the last five years, there were 17 confirmed anthrax cases in the EU/EEA countries, as a result of different activities to combat this disease in both animals and humans.

In this manuscript, we present a large anthrax outbreak that affected both humans and animals in the area of Lonjsko polje in 2022, after a long period of sporadic occurrence in Croatia.

## 2. Anthrax in Croatia

Croatia is a Mediterranean country, located in Southeastern Europe, and like other countries in this part of Europe has an epidemiological pattern of sporadic anthrax occurrences in endemic areas. Reporting is one of the special measures for the prevention and control of infectious diseases, along with early detection of the source of infection and ways of ending transmission, as well as laboratory testing, and is clearly defined by law. In Croatia, there is a long history of anthrax reporting, with the first officially reported human case in the registry dating back to 1945. The incidence of anthrax in humans has been steadily declining and low in recent years, with only two probable cases of cutaneous anthrax reported in the period from 2017 to 2021 [8]. In the past twenty years, cases have occurred sporadically, with a peak in 2005 including 10 recorded cases, but from different parts of Croatia.

Due to the development of veterinary institutional services and the implementation of livestock vaccination from the mid-20th century onward, during the last several decades domestic animal anthrax has been a rare and endemic disease. The first description of animal anthrax in the geographic territory of today’s Croatia originates from 1785 and describes cattle disease in northern parts of the country in Krapina-Zagorje County [9,10]. The regulation on obligatory reporting of anthrax in animals is incorporated in the Croatian Animal Health Law and special Rulebook on reporting animal diseases aligned with Regulation (EU) 2016/429, Commission Implementing Regulation (EU) 2020/2002 and Commission Implementing Regulation (EU) 2018/1882 [11]. It defines regulations for reporting suspected and laboratory-confirmed cases, surveillance dynamics, obligations on animal owners, veterinarians, and diagnostics laboratory reporting, as well as the inspection of the entire process involved in outbreak prevention and control. Regular annual reports from 2009 until 2022 are published and publicly available on the Ministry of Agriculture website [12].

Today, of the 21 Croatian counties, 7 have municipalities known as “anthrax districts” with confirmed sporadic cases. These endemic areas and their boundaries are under specific legislative protection, where default guidelines for control and prevention measures against anthrax have to be implemented.

Until the end of 2021, 27 municipalities in seven different counties have been declared “anthrax areas” with mandatory anthrax vaccination of cattle, sheep, goats, and horses. Sisak-Moslavina County was the region with the most local areas proclaimed as “anthrax districts”. During the past twenty years, two epizootic outbreaks occurred in this county, one among sheep in Jasenovac in 2002 and another among cattle in Sunja in 2006/2007. These two localities are small municipalities situated on the outskirts of Lonjsko Polje Nature Park and are contact zones of park borders. They are located on the opposite bank of the Sava River from the localities where the new anthrax cases occurred in 2022. Besides these epizootics, sporadic animal cases were reported in 2003, 2004, 2005, 2012, and 2014, but were spread over different areas of Croatia [13].

**Figure 1:**
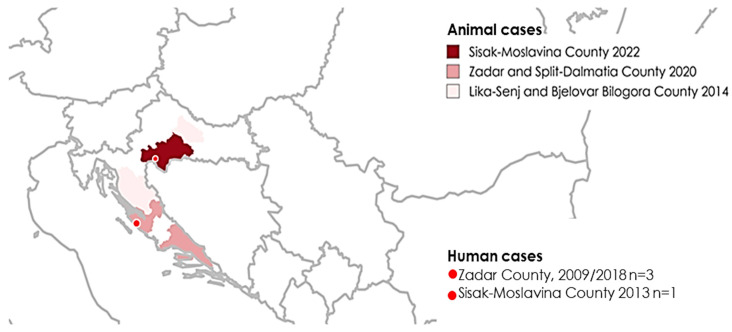
Geographic distribution of counties with anthrax cases in animals and humans in Croatia, 2009–July 2022.

Counties with confirmed anthrax cases in animals and humans from 2009 until July 2022 are presented in Figure 1.

## 3. The Anthrax Outbreak in Croatia in 2022

### 3.1. Outbreak in Animals

Although the Lonjsko Polje Nature Park area is a part of Sisak-Moslavina County, its entire territory has never been assigned as an anthrax district in the past. The competent veterinary authority of Croatia received the first information on the suspected anthrax in the Osekovo pasture within Lonjsko Polje Nature Park on 13 July 2022 from an authorized veterinarian. Specimens from the dead cattle were sent for pathohistological examination to the Croatian Veterinary Institute, but due to the advanced autolysis of the carcass, the isolation of *B. anthracis* was not possible.

On 15 July 2022, the death of an additional cattle was reported at the Osekovo pasture, in which the microbiology examination confirmed the *B. anthracis* infection. At the same time, the veterinary inspector referred the animal owner for further medical treatment due to suspicion of cutaneous anthrax.

Although the disease was officially reported to the competent authority for the first time on 13 July 2022, the outbreak most probably started earlier. The increased number of dead cattle and horses in the stage of carcasses or decomposition suggested a possible start during May and June. A lack of early response occurred as a result of the animal owner’s legally non-compliant behavior and disobedience of the legislation linked to the disease notification obligation (to report suspicions of animal disease). From the very beginning of the epidemic, animal owners were continuously educated by the competent veterinary services about the importance of reporting deaths and how to deal with animals and their carcasses in the infected area.

**Figure 2:**
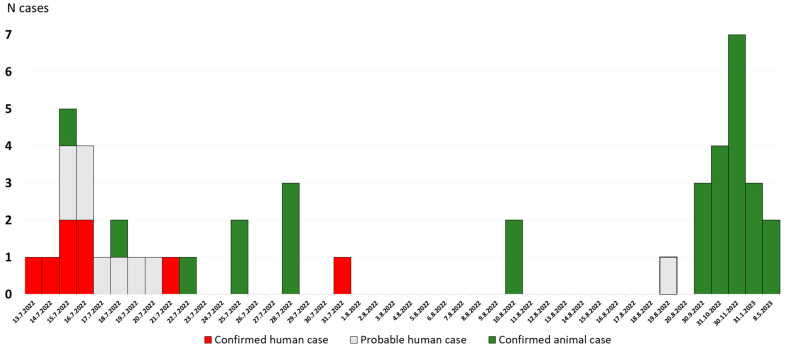
Timeline of human and animal anthrax cases during the outbreak.

**Figure 3:**
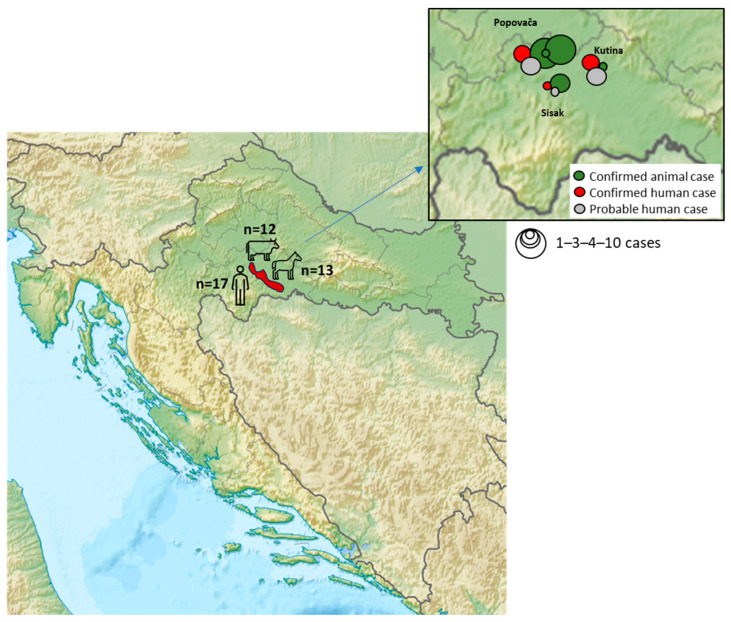
Geographic distribution of human and animal anthrax cases during the outbreak. Red shadowed color represents the Lonjsko Polje area, where the outbreak occurred.

In the period from 13 July 2022 to 15 May 2023, when the last positive animal case of the disease was confirmed, a total of 29 positive anthrax cases were confirmed by bacteriological examination of dead animal samples (17 in horses and 12 in cattle) (Figure 2). The majority of cases were reported in 2022 (*n* = 24), while in 2023 there were only five cases of anthrax in domestic animals. Positive animals were found dead, and therefore, it was not possible to determine the disease onset or the clinical manifestations. The animals were distributed on common pastures and establishments (holdings) located in Osekovo (Kutina City), Gračenica (Popovača City), Repušnica (Kutina City), and Veliko Svinjičko (Sisak City) (Figure 3). Due to issues in reporting deaths in animals, there are assumptions that the number of animals affected by the disease was much higher.

The Order on measures to prevent the occurrence and spread of anthrax in the infected area was issued on 22 July 2022 by the Croatian Ministry of Agriculture [14]. To control the outbreak, the competent veterinary authority determined a number of activities and measures on the pastures in the area of the Lonjsko Polje Nature Park, with continuous cooperation with all relevant stakeholders and services (veterinary inspections, veterinarians, animal owners, ranger service of the Lonjsko Polje Nature Park, representatives of local government units, civil protection, hunting associations, police, and medical service). By the aforementioned Order, the area of common pastures of the Lonjsko Polje Nature Park was declared an infected anthrax area, and all livestock transport in and out of the affected area was prohibited. Moreover, obligatory reporting was issued for all suspected cases and locations of domestic and wild animals’ carcasses. Vaccination of all cattle, sheep, goats, and equines was declared obligatory, including livestock that had resided in the affected area and been shipped away previous to the outbreak. Vaccination of susceptible animals in these areas will be carried out continuously every year as an obligatory measure until the “anthrax district“ is ruled out. Moreover, several prohibition measures were implemented, including prohibition of entrance inside the affected area for all, except experts and persons with special veterinary inspection permits; prohibition of hunting and trading with meat products; and prohibition of all agricultural activities, including forest activities and grass mowing. In addition, obligatory disinfection was ordered for field wells and vehicles passing through the affected area.

### 3.2. Outbreak in Humans

The first human case was suspected on 15 July 2022 in a young patient with cutaneous presentation. Swab samples were collected from the skin lesions for laboratory confirmation of *B. anthracis* infection. Two sterile synthetic (Dacron) swabs were used for aseptic sample collection (direct microscopy and bacterial cultivation) and were sent to the local microbiological laboratory at the Public Health Institute of Sisak-Moslavina County and to the Microbiology service of the Croatian Institute of Public Health. Microscopy of swab smears revealed gram-positive bacilli, single and in chains in the formation of bamboo sticks. The culture showed dry, gray, slightly convex with a rough surface, nonhemolytic, rapidly growing colonies, 3–5 mm in diameter, after overnight incubation at 37 °C. The isolate was catalase-positive and non-motile. Although *B. anthracis* was suspected, the final identification was challenging. In the further process of identification, an automated biochemical analysis of suspicious colonies on a VITEK 2 device (Biomerieux, Marcy-l’Étoile, France) using a Bacillus identification card for the identification of aerobic endospore-forming bacteria was performed. The VITEK result was *Geobacillus toebii* 99%. The *B. anthracis* was confirmed by PCR identification of grown colonies. DNA extraction was performed using the QIAamp DNA Mini Kit (QIAGEN, Venlo, The Netherlands). For PCR testing, the *Bacillus anthracis* Genesig^®^ Standard kit (Primerdesign™ Ltd., Eastleigh, UK) was used.

In the next several weeks, more patients presented with similar cutaneous lesions, the last reported on August 19 (Figure 2 and Figure 3). All persons who were in contact with the infected livestock were contacted by epidemiologists. Epidemiological activities among others involved: referring patients with suspicion of disease to an infectious disease specialist; educating the population about the symptoms of cutaneous anthrax; prescribing chemoprophylaxis to healthy individuals who had been in contact with infected livestock; advising against consuming the meat from dead livestock; and emphasizing the importance of wearing protective equipment when working with livestock. The cutaneous form of anthrax was recorded in 17 patients. The skin lesions were mostly located on the exposed body parts: hands, forearms, and shins. The lesions were presented in different stages, including red papules, ulcerations with surrounding vesicles, and peripheral edema and eschar formation. The laboratory confirmation was achieved in eight patients, while the remaining nine patients presented with late-stage changes, making bacteriological confirmation unlikely. All patients had a history of contact with infected or dead animals. Among the patients, three were children up to 9 years old, six were teenagers in the 15 to 18 years age group, and eight were adults. All patients were treated with antibiotic therapy and fully recovered. Moreover, 94 healthy individuals who were in contact with infected livestock received chemoprophylaxis. The last human case was detected on 19 August 2022. Since then, not a single suspected or confirmed case of the disease has been identified (Figure 2).

### 3.3. Geographic Characteristics of New Anthrax Areas in the 2022 Outbreak

Lonjsko Polje Nature Park and its settlements cover 506.5 square kilometers of protected wetland area, the largest wetland area in Croatia and the Danube basin. It is situated longitudinally along the river Sava and its six minor tributary rivers. Due to the composition of the soil, the dedicated agricultural activity in the Park is livestock breeding. Pastures and grassland are used for cattle, swine, and horse traditional grazing, meaning that animals live in the open day and night 24 h a day from spring to winter with occasional human supervision [15].

The Lonjsko Polje area belongs to the phreatic type of humid climate. The recorded water levels of the river Sava’s watercourses in June and July 2022 were extremely low due to longer dry periods, especially in the area near Lonjsko Polje (Jasenovac measuring station). Water levels were lower by more than 74% in June and by 50% in July compared to the average of the last 30 years (1991–2020) [16,17]. The soil in the park is composed of mixed mineral and non-mineral, with carbonate predominating in mineral soil. Based on the seismic-tectonic characterization of the Lonjsko Polje area and the nearby Sisak-Moslavina County area, the expected maximum earthquake magnitudes were VI-VII (Modified Mercalli intensity scale; MCS). However, on 29 December 2020, an earthquake hit with the intensity in the epicenter of VIII (MCS), and numerous weaker aftershocks followed during 2021. The distance between the earthquake epicenter and Lonjsko Polje is about 30 km. After the earthquake, the phenomena of soil collapsing and the formation of 122 new cover-collapse sinkholes ranging from 1–25 m in diameter and 0.2–11 m in depth, filled with water and vegetation, occurred in the villages of Mečenčani and Borojevići in Sunja River Valley, which is one of the Lonjsko Polje Nature Park tributary rivers situated on its south border [18]. These villages are part of the “anthrax district” and are about 50 km away, separated by the Sava River from the pasture of Osekovo on the north border of Nature Park, where the first cases of animal disease were reported.

## 4. Discussion

The outbreak described in this report represents the largest anthrax outbreak that has been documented in Croatia and the EU for the past 30 years. Due to the coordinated action of all stakeholders involved, both human and veterinary medicine specialists, the disease was successfully put under control. Since January 2023, no new animal and human cases have been observed. Several possible factors might be drivers of this outbreak. First, the high-magnitude earthquake that hit the area could influence the movement and rising of soil containing infectious spores from the deeper layers of the earth to the surface. Moreover, the seismic activity could redirect groundwater flows to new areas in the vicinity of well-known anthrax districts. In addition, the occurrence of new and enlargement of old sinkholes after the earthquake lasted until the end of 2021, possibly affecting spores’ dissemination. The ability of *B. anthracis* to form spores, its life cycle, and survival time depend on the soil composition and meteorological and environmental conditions. Larger concentrations of spores are found in soils having a slightly alkaline pH, higher organic matter, and higher calcium content, as was the case with the soil composition in Lonjsko Polje. These favorable soil characteristics enable the bacterium to have a longer latency and survival [19].

The weather conditions during 2022 may have also affected the magnitude of the presented outbreak. The Lonjsko Polje area experiences extensive flooding during spring, which can bring spores closer to the surface due to the rise of underground water levels. However, due to the extreme reduction of the Sava River water flow along the entire watercourse through Lonjsko Polje in June and July 2022, flooded areas dried up otherwise, allowing pastures to expand and providing opportunities for cattle and horses to graze. Spores’ ability to concentrate due to their high surface hydrophobicity, along with their additional concentration during water evaporation, enable them to be collected and carried by water in “storage areas”, thus contaminating grass and other vegetation [20,21]. Conditions such as the hot and dry summer season with extreme wetland draining significantly influenced the type of animal food intake. Namely, the animals were forced to graze the plants by pulling them off the soil surface due to poor, low-quality grazing. Such ingestion of hard and dry herbs could cause mouth mucosal damage and facilitate the contact and ingestion of spores from the soil.

In addition, vaccination of livestock was not obligatory, since areas and settlements where the outbreak occurred have not been declared to be “anthrax areas”. Having no experience with anthrax, farmers were not fully aware of the disease and how to handle sick animals, leading to many infections in humans. Without a rapid response starting from the first animal case, soil contamination by infected carcasses and transmission of spores by scavengers coming and leaving the area was undisrupted for a prolonged period.

After clinical suspicion of a possible anthrax case, fast and accurate laboratory identification is of paramount importance for therapy management. Due to the high genetic similarity of *B. anthracis* with other species of the *B. cereus* group, it is very important to emphasize that rapid identification with automatic devices such as VITEK 2 and MALDI-TOF may be challenging. During bacterial identification, particular caution should be paid to conduct accurate species identification in order to avoid false positive or false negative results.

Of the 17 reported human cases, only 8 had laboratory confirmation due to late-stage skin lesions. Farmers rarely seek medical help for lesions and/or symptoms with mild presentations. Moreover, because of the lack of experience with anthrax lesions and the knowledge of possible disease progression, they were unaware of the possible health risks. It is important to point out that despite the later appearance of cases in animals, which were recorded until the spring of 2023, not a single case of human disease occurred after August 2022. We believe that this is the result of compliance with the measures introduced in prevention and outbreak control.

There is a limitation of the study that needs to be addressed. Genotyping data of *Bacillus anthracis* samples of the reported outbreak were not available; therefore, it was not possible to show if the outbreak victims were affected by closely related bacterial species.

## 5. Conclusions

Collaboration based on timely and open communication between all participants involved in outbreak detection and control, from farmers to local, regional, and state authorities, has stopped the spread of the outbreak and enabled lessons to be learned for future emerging challenges. The assurance of quality control in veterinary and human medical procedures, as well as ones in administrative institutions in charge of surveillance, control, and legislation, must be strictly implemented, continuously followed up, and controlled.

Additionally, the occurrence of natural disasters, such as earthquakes, floods, and drought, requires that one consider expanding preventive measures to new potential risk areas in zones close to previously affected districts in order to prevent the emergence and re-emergence of important zoonotic pathogens.

Continuous awareness, preparedness, and rapid action in case of novel biological threats of all services included in the prevention, diagnostics, and treatment of animal and human anthrax is important for the protection of public health. Through the coordination of veterinary and medical professionals, the largest outbreak in Croatia, and also in the entire EU, in the last 30 years has been controlled, confirming the importance of the “One Health” concept in the prevention of zoonotic diseases.

## Data Availability

Data are contained within the article.
